# Occurrence of Colibacillosis in Broilers and Its Relationship With Avian Pathogenic *Escherichia coli* (APEC) Population Structure and Molecular Characteristics

**DOI:** 10.3389/fvets.2021.737720

**Published:** 2021-09-08

**Authors:** Ilias Apostolakos, Andrea Laconi, Lapo Mughini-Gras, Özlem Şahan Yapicier, Alessandra Piccirillo

**Affiliations:** ^1^Department of Comparative Biomedicine and Food Science, University of Padua, Legnaro, Italy; ^2^Center for Infectious Disease Control, National Institute for Public Health and the Environment (RIVM), Bilthoven, Netherlands; ^3^Faculty of Veterinary Medicine, Utrecht University, Utrecht, Netherlands; ^4^Republic of Turkey Ministry of Agriculture and Forestry Veterinary Control, Central Research Institute, Bacteriology Diagnostic Laboratory, Ankara, Turkey

**Keywords:** avian pathogenic *E. coli* (APEC), broiler-chicken, WGS (whole genome sequencing), antimicrobial resistance (AMR), avian fecal Escherichia coli (AFEC), ST11689, colibacillosis, IncF plasmid

## Abstract

Avian pathogenic *Escherichia coli* (APEC) causes colibacillosis, the disease with the highest economic loss for the broiler industry. However, studies focusing on the prevalence and population structure of APEC in the broiler production pyramid are scarce. Here, we used genotyping and serotyping data to elucidate the APEC population structure and its changes in different broiler production stages along with whole-genome sequencing (WGS) in a subset of APEC isolates to determine transmission patterns amongst dominant APEC sequence types (STs) and characterize them in detail. Comparison of genotypes encountered in both APEC and avian fecal *E. coli* (AFEC) provided further insights. Overall, APEC-related mortality, as the proportion of the total sampled mortality in the broiler production, was high (35%), while phylogroup C and serogroup O78 were predominant amongst APEC isolates. We found a low (34.0%) and high (53.3%) incidence of colibacillosis in chicks and end-cycle broilers, respectively, which may be related to a shift in APEC genotypes, suggesting a trend from commensalism to pathogenicity across different broiler production stages. Despite considerable APEC genotypic diversity, there was substantial genotype overlap (40.9%, overall) over the production stages and convergence of STs to the four clusters. Within these clusters, WGS data provided evidence of clonal transmission events and revealed an enriched virulence and resistance APEC repertoire. More specifically, sequenced APEC were assigned to defined pathotypes based on their virulence gene content while the majority (86%) was genotypically multi-drug resistant. Interestingly, WGS-based phylogeny showed that a subset of APEC, which are cephalosporin-resistant, may originate directly from cephalosporin-resistant AFEC. Finally, exploration of the APEC plasmidome indicated that the small fraction of the APEC virulome carried by IncF plasmids is pivotal for the manifestation of the APEC pathotype; thus, plasmid exchange can promote pathogenicity in strains that are at the edge of the commensal and pathogenic states.

## Introduction

*Escherichia coli* is a commensal facultative anaerobic microorganism that inhabits the lower gastrointestinal tract of mammals and birds shortly after birth and acts as a symbiont involved in the synthesis of necessary vitamins for their hosts (1). As a versatile microorganism characterized by genomic plasticity, *E. coli* can enrich its accessory genome with virulence genes (VGs) that enable its adaptation to unfavorable environmental conditions and the colonization of extra-intestinal organ niches that can lead to adverse health effects for their hosts (2). Several *E. coli* lineages evolved preserving genomic regions where VGs accumulate, termed pathogenicity islands. Other VGs are fixed on large, transmissible plasmids (mainly of incompatibility group IncF) that increase the virulence potential of recipient *E. coli* isolates (3). Depending on the organs in which pathogenic *E. coli* usually exert their virulence, they are separated into two main groups; intestinal pathogenic *E. coli* (InPEC) and extra-intestinal pathogenic *E. coli* (ExPEC) (4). A subset of the latter group, avian pathogenic *E. coli* (APEC), is a particularly important pathotype for the broiler industry since it causes colibacillosis, a disease with significant economic losses due to the mortality and/or reduced productivity of affected birds. The clinical manifestations of colibacillosis range from yolk sac infection (omphalitis) in broiler chicks and septicemia in broiler chicks and adults, to reproductive tract infection (salpingitis) in layers. Respiratory infection (airsacculitis) is widespread in both broilers and layers (4, 5), and lesions in other visceral organs (perihepatitis, pericarditis) are also encountered (5). Common antigenic and genotypic characteristics of APEC in broilers include serotypes O1, O2 and O78, phylogroups B2 and D, and multilocus sequence types (STs) of the ST23 and ST95 clonal complexes (6). However, clinical APEC isolates recovered from cases of colibacillosis are far from limited to these characteristics and usually show exceptional diversity between countries and even within the same flock or disease outbreak, representing a challenge for the prompt diagnosis and prevention of the disease (5). The VG content of APEC (and ExPEC) is an additional conundrum since specific virulence determinants have not been explicitly associated with colibacillosis and some of those more frequently identified in pathogenic isolates have also been recovered from their commensal counterparts (i.e., avian fecal *E. coli*, AFEC). Indeed, Kemmett et al. (7) showed that the intestine of day-old chicks was rich in AFEC with pathogenic potential and thus it constituted a reservoir of APEC and VGs thereof. Conversely, the recovered clinical isolates belonged to “commensal” phylogroups (e.g., A and B1) and/or lacked APEC-indicating VGs. These findings highlight our incomplete understanding of this important pathotype (8).

Notwithstanding the economic impact of colibacillosis in the poultry industry, there is only a handful of investigations considering the whole broiler production pyramid in the determination of the prevalence, molecular characteristics and population structure of APEC. Here, we addressed this knowledge gap by elucidating the APEC structure and modeling its population shifts in subsequent broiler production stages with a combination of basic and high-resolution methods. Our approach enabled us to gain concrete evidence of clonal and horizontal transmission events of dominant APEC STs, as well as detailed characterization of their resistance, virulence arsenal, and IncF plasmidome. Further insights were obtained from the juxtaposition of STs encountered in both the APEC and AFEC populations of the studied broiler flocks.

## Materials and Methods

### Sampling Design

Three generations of broilers (production chains A, B, and C) from an integrated broiler company in Italy were longitudinally followed and sampled. Sampling was conducted in conjunction with that of another study that determined the rate of cephalosporin resistance in randomly selected AFEC isolates from apparently healthy broilers (9, 10). The samples of this study consisted of dead standard commercial broilers collected during the first daily welfare walk on visited farms and dead-on-arrival (DOA) broilers at the slaughterhouse. Briefly, we first sampled one parent flock (PF) per production chain at ~21 weeks of age during the laying period. Due to biosecurity restrictions, only a limited number of samples was obtained from PFs. The offsprings of the PF were sampled in four fattening farms per production chain (twelve in total) at two time points; at the start (~1–2-day-old broiler chicks) and the end (~30-days-old broilers) of the production cycle, sampling the same house (shed) of the farm at both time points. Furthermore, DOA birds originating from the previously sampled broiler flocks were collected at the slaughterhouse after the unload of the transportation truck. During sampling, information on the number of housed birds and mortality rates was retrieved from the responsible veterinarians.

### Post-mortem Examination and *E. coli* Isolation

Dead birds were subjected to post-mortem examination within the same day of sampling except for those from two farms, which were first stored at −20°C for 2 days. Only recently dead birds showing no signs of injury or having flattened appearance were subjected to post-mortem examination to avoid contamination of tissue samples with bacteria originating from ruptured intestine. Classical signs of colibacillosis (e.g., airsacculitis, perihepatitis, pericarditis) were recorded. Visceral organs (i.e., spleen, brain, liver, lungs, air sacs, yolk sac, oviduct) and pericardium were aseptically sampled with sterile swabs upon sterilization of the organ surface with a burning hot iron spatula, when appropriate. Swabs were directly streaked on Eosin Methylene Blue (EMB) agar (Microbiol, Italy) and incubated overnight at 37°C. One to two morphologically typical *E. coli* colonies on EMB (metallic green sheen) were isolated from each sample and stored at −80°C in 20% glycerol.

### Detection of Resistance to 3rd Generation Cephalosporins (3GCs)

All *E. coli* isolates were screened for resistance to 3GCs and production of extended-spectrum beta-lactamases (ESBLs) by double-disk synergy test using cefotaxime (30 μg) and ceftazidime (30 μg) discs with and without clavulanic acid (10 μg) and according to CLSI guidelines (11). Additionally, a cefoxitin disc (30 μg) was used to detect potential AmpC beta-lactamase (pAmpC)-producers.

### Serological and Molecular Characterization

Serogroups were determined by standard slide agglutination test with antisera (Star Ecotronics, Italy) against O-somatic antigens for at least one isolate per bird diagnosed with colibacillosis (*n* = 95 were tested) in two phases; first, isolates were tested against the most common APEC serogroups, namely the O1, O2, and O78. Those found negative were sent for testing with an extended panel of common APEC antisera at an external service (Istituto Zooprofilattico Sperimentale della Lombardia e dell' Emilia Romagna, Italy).

All isolates (*n* = 153) were subjected to multiplex PCRs for species confirmation and determination of *E. coli* phylogroups using the updated Clermont protocol (12). To further explore the genetic diversity of isolated APEC, selected isolates (*n* = 38) were genotyped for their multilocus sequence types (STs) and clonal complexes (ST Cplx) according to the Achtman scheme (13). This selection was done considering the diversity of *E. coli* phylogroups and serogroups in our dataset and at least one serogroup-phylogroup combination per production stage was genotyped. Alleles' sequences for each isolate were concatenated, aligned with MAFFT (14) and a maximum-likelihood (ML) phylogenetic tree was built with IQ-TREE (14) with 1,000 bootstraps. Additionally, hierarchical clustering was performed with the hierBAPS module of the Bayesian Analysis of Population Structure (BAPS) software v6.0 (15). In phenotypically 3GC-resistant *E. coli*, ESBL/pAmpC genes were amplified (16) and sequenced (Macrogen, Spain). Analysis for chromosomal mutations in the *ampC* promoter/attenuator was performed as well (17).

### Whole Genome Sequencing and *in silico* Typing

In order to find evidence of clonal and horizontal transmission events across the broiler production pyramid, as well as gain insights into the complete resistance and virulence profile of APEC, a selection of 23 isolates was subjected to whole-genome sequencing (WGS). This selection included isolates of the dominant STs (ST23, ST117, ST371, ST744, and ST1485) (*n* = 17), which had at least two isolates in the collection, and six isolates belonging to singleton STs. The WGS data set was coupled with 18 previously sequenced AFEC (9) isolates from the same broiler flocks, belonging to the dominant APEC STs and showing phenotypic and genotypic resistance to 3GCs (AFEC-ESBL). For all isolates, DNA was isolated with the Invisorb Spin Tissue Mini Kit (Invitek, Germany), library preparation was done with the Nextera XT library preparation kit (Illumina, USA) and sequenced on an Illumina HiSeqX platform with 2 × 100 bp paired-end reads at a private company (Macrogen, Korea). Raw reads were directly submitted to the Enterobase database (18) for read pre-processing and SPAdes (19) assembly and were deposited in the Sequence Read Archive (SRA) with BioProject accession number PRJNA745070Assembly statistics (coverage, N50, genome size, and contig number) and accession numbers of assemblies (Enterobase barcodes) for each strain can be found in Supplementary File 1. *E. coli* STs were predicted through the Enterobase pipeline, which follows the Achtman scheme. Moreover, APEC genomes were *in silico* genotyped for acquired resistance genes (RGs) with ResFinder 4.0 (20), plasmid replicon types (PlasmidFinder 2.1) (21) and plasmid STs (pMLST 2.0) (21), using the tools with default settings. The virulence gene (VG) profile and resulting pathotype of APEC were assessed with alignments to the Virulence Factors Database (VFDB) (22) using the MyDbFinder tool (https://cge.cbs.dtu.dk/services/MyDbFinder/). To investigate a possible correlation between VGs, STs, and phylogroup a hierarchical clustering including APEC and AFEC-ESBL was generated using the frequency of the presence of each virulence gene, scored as binary data (present = 1, absent = 0) in R version 4.1.0 (https://www.r-project.org/). Additionally, plasmids were identified with the MOB-suite (23) tool and were separately screened with ResFinder and MyDbFinder to determine the genomic localization (chromosomal or plasmidic) of the identified RGs and VGs. Our plasmid analysis primarily focused on IncF plasmids, which are known to harbor crucial APEC VGs and thus play a pivotal role in APEC pathogenicity (5). To infer the phylogeny of whole-genome sequenced APEC, a maximum likelihood single nucleotide polymorphism (SNP) tree was created with CSI phylogeny (24) using the default settings. The IncF plasmids, predicted with MOB-suite, were analyzed for their core and accessory genome with the PIRATE (25) toolbox. The phylogeny of IncF plasmids was inferred with IQ-TREE using the alignment of the core plasmid genome as input. Part of the WGS analysis was done on the European public Galaxy (26) server (https://usegalaxy.eu/).

### Data Analysis

The occurrence of colibacillosis amongst dead commercial broilers, both overall and for each production stage, was calculated based upon organ samples (at least one) with typical colibacillosis lesions being positive for *E. coli* colonization after post-mortem examination. Prevalence estimates and corresponding 95% confidence intervals (95% CI) were adjusted for clustering of observations at the chain and farm levels using cluster-robust standard errors. Significance of differences in the occurrence of colibacillosis in different stages was tested using logistic regression analysis accounting for chain and farm clustering. The distributions of phylogroups in different production stages between the APEC of this study and the AFEC of our previous investigation (10) were assessed with chi-square test for equality of proportions to identify differences in the enrichment of specific phylogroups between the two *E. coli* populations. Moreover, chi-square statistic for trends in proportions was used to test the significance of trends in the relative frequencies of different phylogroups and serogroups over subsequent sampling stages across the whole broiler production pyramid. To quantify transitions in the APEC population across the broiler production pyramid, the average pairwise overlap of the phylogroup-serogroup feature was estimated based on the proportional similarity index (PSI) (27), accounting for uncertainty in measurements in a Monte Carlo simulation setting. PSI values range from 0 (no similarity) to 1 (total overlap). Moreover, the Simpson's index (28) (SI) of diversity was used to measure the diversity of APEC in terms of phylogroups and serogroups per production stage as the probability that two strains randomly selected from a given sampling stage would belong to different phylogroups or serogroups. Statistical analyses were performed using STATA (StataCorp, College Station, USA).

## Results

### Post-mortem Examinations

Three breeder flocks and twelve broiler flocks were sampled. The breeder flock size was 28,956 ± 9,531 (mean ± standard deviation), and a high mortality (9.4 ± 3.9%) was recorded due to salpingitis outbreaks in two of the three breeder flocks. The broiler flock size was 24,033 ± 9,909 with a total end-cycle mortality of 5.47 ± 1.0%. Overall, 144 dead broilers were subjected to post-mortem examination and 50 thereof (35.0%, 95% CI 20.4–53.0%) were found having typical lesions of colibacillosis and *E. coli* isolates were recovered from their organs. In total, 153 *E. coli* isolates were retrieved from 50 birds with clear lesions of colibacillosis (a median of three isolates per bird was analyzed). They were isolated from eleven different organs, primarily the liver (perihepatitis, 20.9%), pericardium (pericarditis, 18.3%), and the air sacs (airsacculitis, 17.0%). Other frequent infection sites were the yolk sac (omphalitis, 10.5%), the brain (encephalitis, 7.2%), and the subcutaneous tissues (cellulitis, 7.2%). *E. coli* associated mortality (salpingitis/peritonitis) was high (83.3%, 95% CI 5.4–99.8%) for the PFs, however the number of samples for this production stage was small (*n* = 7). About one-third (*n* = 53, 34.0%, 95% CI 24.3–45.2%) of the mortality in broiler chicks could be attributed to *E. coli* infection, whereas for broilers in the last week of life as well as DOA broilers the ratio were 27.5% (n=69, 95% CI 4.2–76.6%) and 53.3% (*n* = 15, 95% CI 37.0–69.0%), respectively. There were no significant differences in the occurrence of colibacillosis between production stages.

### Molecular and Serological Characterization

**Figure 1:**
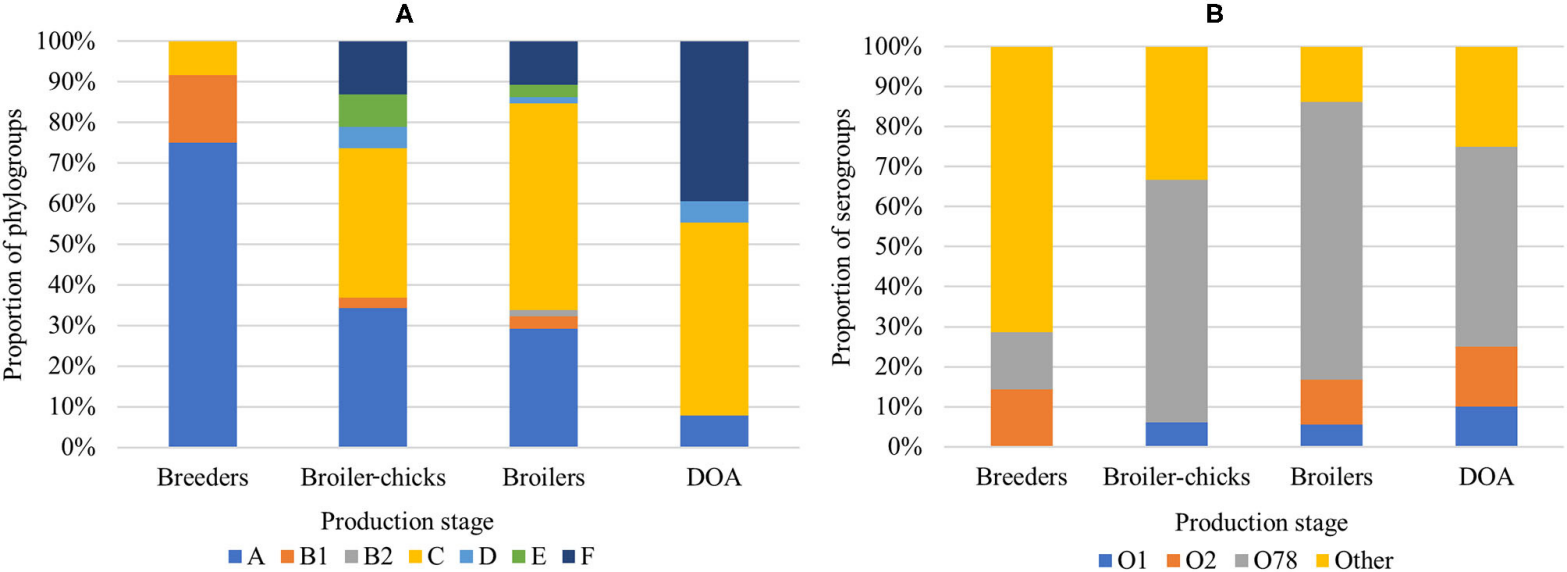
Distribution of **(A)** APEC phylogroups and **(B)** APEC serogroups in the broiler production pyramid.

In the phylogenetic analysis, APEC were predominantly assigned to phylogroup C (43.1%) followed by A (28.8%) and F (17.6%). Other phylogroups (B1, B2, D, and E) were altogether less frequent (9.8%) (Figure 1A). In half of the colibacillosis cases (*n* = 25) isolates from the same bird were assigned to multiple phylogroups, suggesting the possibility of multi-strain infections from diverse APEC genotypes in these birds. Regarding their O-somatic antigens, the majority of isolates were assigned to the prominent APEC serogroup O78 (58.9%) but also to O2 (8.4%) and O1 (6.3%). Each of the other identified serogroups (i.e., O8, O23, O45, O55, O89, O103, O149, and O161) individually occurred in <3% of isolates, meanwhile 11.6% were non-typeable (NT) by the slide agglutination test.

### Data Analysis

Significant relationships of the production stage with the isolation organ, phylogroup and serotype (*p* < 0.05) were revealed, while SI values provided an estimation of APEC homogeneity in each stage. In breeders, the majority (91.7%) of isolates was collected from lesions of ovaritis/salpingitis. In this production stage, we encountered largely homogenous phylogroups (SI 44%) with group A being predominant (75.0%) and the largest serological diversity (SI 95%) with seven isolates being distributed in six serogroups (Figure 1B). APEC from broiler chicks were primarily isolated from omphalitis (42.1%) but also from peripheral organs such as the liver (23.7%) and the brain (21.1%). Here, the largest phylogenetic diversity (SI 74%) with groups C and A being predominant (36.8 and 34.2% of APEC, respectively) was observed, while serotypes were notably more uniform (SI 63%) due to the abundance of O78 isolates (60.6%). In end-cycle broilers sampled at the farm and as DOA birds, the most common lesions were those of pericarditis, perihepatitis, and airsacculitis, while APEC isolates were phylogenetically less diverse compared to broiler chicks (Figure 1A). Of note, there was a gradual but significantly increasing frequency of group C (linear slope = 0.1, SE = 0.04, *p* < 0.0001) and F (linear slope = 0.17, SE = 0.04, *p* < 0.0001) along the production chain, which eventually displaced the decreasing group A (linear slope = −0.19, SE = 0.04, *p* < 0.0001) in broilers and DOA broilers. In terms of serogroups, predominance of the O78 was maintained (69.4 and 52.6% in broilers and DOA broilers, respectively) but with an apparent increase of O1 and O2 (Figure 1B). An additional analysis was performed to compare the distributions of phylogroups in different production stages between the APEC and AFEC populations. Significant differences in the phylogenetic background of the two populations were found for each production stage (Supplementary Figure 1). Moreover, phylogroup C was more enriched in APEC, whereas phylogroups E, B1, and B2 were more represented in AFEC.

The APEC phylogroup-serogroup combination was used as an additional APEC feature to calculate the PSI and estimate shifts in the APEC population structure in subsequent production stages along the production chain. The average overlap of phylogroup-serogroup distributions over all sampling points was 40.9%, denoting an overall heterogenous APEC population. The smallest overlap was observed between breeders and broiler chicks (Table 1). In contrast, we estimated an overlap of 55.5% of genotypes found in broilers and broiler chicks. A small but notable increase was observed in the overlap between APEC isolates from end-cycle broilers at the farm and DOA broilers at the slaughterhouse (Table 1).

**Table 1:** Simpson's Index (SI) and proportional similarity index (PSI) for APEC in different sampling points.

**Production stage**	**Simpson's index**		**PSI**
	**Phylogroups %**	**Serotypes %**	Phylogroups* **Serotypes^**a**^%**
Breeders	44	95	-
Broiler chicks	74	63	7.3
Broilers	65	50	55.5
DOA	63	70	59.9
Overall	90	87	40.9

a *Values represent % occurrence of combined serotype and genotype overlap between subsequent production stages*.

### MLST Phylogenetic Analysis

**Figure 2:**
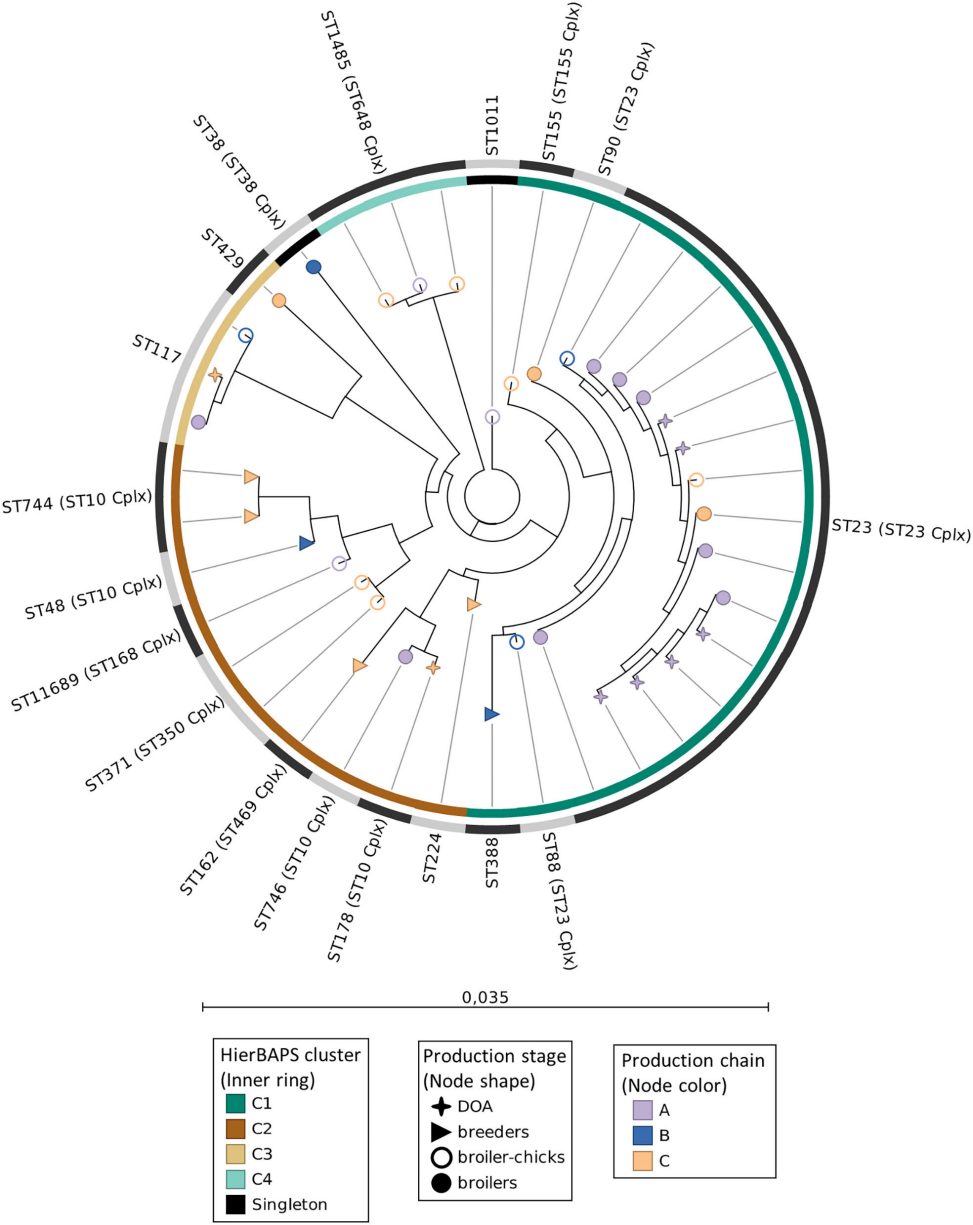
Maximum-likelihood phylogeny of APEC MLST sequences. Nodes' shape and color indicate the broiler production stage and production chain, respectively. The inner ring specifies the hierBAPS clusters. The outer ring with alternating dark and light gray segments, marks different STs. Scale bar refers to the branch lengths, which are measured in the number of substitutions per site.

Analysis by MLST revealed a phylogenetically diverse APEC collection as 38 typed isolates were assigned to 18 distinct STs (Figure 2). The most prevalent ST was ST23 (ST23 Cplx) (39.5% of APEC) recovered from broiler chicks, broilers, and DOA broilers (Figure 2), followed by the equally represented ST1485 (ST648 Cplx) and ST117 (7.9% each). Except for ST371 (ST350 Cplx) and ST744 (ST10 Cplx) (5.3% each), all the other STs (*n* = 13) occurred as singletons. The phylogroup-serotype attribute proved to be an adequate indicator of APEC diversity given that different phylogroup-serotype combinations were generally assigned to different STs (Supplementary File 1). The first isolate of a novel ST, ST11689 (ST168 Cplx), was identified for the first time in this study, recovered from a broiler chick of chain B. This novel ST is closely related to ST93 (ST168 Cplx) as they differ by only one SNP in the *icd* allele (C232T, icd10 → icd1435). Cluster analysis with hierBAPS revealed the presence of four clusters (C1–C4) and two singleton isolates (Figure 2). The largest cluster C1 (*n* = 19, 50% of typed isolates) mainly comprised all isolates of the dominant ST23 and other STs of the ST23 Cplx (ST90 and ST88) as well as ST155 and ST388, with which ST23 shares two and three MLST alleles, respectively. The dominance of C1 is evidenced by the successful spread of this cluster across production chains, although mainly chain A, and production stage as depicted in Figure 2. The second largest cluster C2 was notably more diverse given that it included eight different STs, which however mainly belonged to the ST10 Cplx. Of interest was the displacement of ST10 Cplx isolates by isolates belonging to other clonal complexes in the ML phylogenetic tree. ST10 is the largest and more diverse clonal complex and includes STs retrieved from various niches (26). Moreover, while the MLST scheme relies on allelic differences (locus variants) to assign STs in clonal groups, the ML phylogeny is sequence-based. Although ST10 Cplx isolates shared more alleles in the ML tree, they were displaced by STs of different complexes with which several ST10 Cplx isolates had less SNP differences (data not shown). Moreover, cluster C2 included the novel ST11689 and was generally more diverse in terms of its dissemination across different production chains and stages. The last two small clusters mainly contained three isolates of ST117 (C3) and ST1485 (ST649 Cplx), respectively.

### Resistance to 3GCs

All APEC isolates were screened for phenotypic resistance to 3GCs by disk diffusion and 10/153 (6.5%) were found positive. In these isolates, we confirmed the presence of ESBL/pAmpC genes by identifying *bla*_CMY−2_ (*n* = 4), *bla*_SHV−12_ (*n* = 3), *bla*_TEM−52_ (*n* = 1), and *bla*_CTX−M−1_ (*n* = 1). Additionally, one isolate had the following mutations in the *ampC* gene control region: −88 (C → T), −82 (A → G), −42 (C → T), −18 (G → A), −1 (C → T), and +58 (C → T).

### Resistance Genes Other Than ESBL/pAmpC

APEC subject to WGS (*n* = 23) carried resistance genes to four antimicrobial classes (min = 1, max = 8) on average, and 86% were multi-drug resistant (MDR; resistance to three or more antimicrobial classes). Aminoglycoside acetyltransferase genes (*aac(3)*-like), nucleotidyltransferase genes (*aadA*-like) and phosphotransferase genes (*strA, strB, aph(3*′*)*-like) were found in 82.6% of isolates and an equal proportion of APEC carried *sul*-like sulphonamide resistance genes. The narrow-spectrum beta-lactamase *bla*_TEM−1b_ gene was present in 73.9% of APEC. Moreover, the tetracycline resistance genes *tet*(A) and *tet*(B) were found in 21.7 and 8.7% of APEC, respectively, whereas trimethoprim resistance, conferred by *dfrA*-like genes, was found in 30.4% of isolates. Plasmid-mediated quinolone resistance (PMQR) genes were identified in 17.4% of APEC, specifically *qnrS1* (8.8%), *qnrS2* (4.3%), and *qnrB19* (4.3%). Quinolone resistance was also mediated by chromosomal mutations in the quinolone resistance determining regions (QRDR) in 47.8% of isolates, with the most prevalent mutations resulting in the amino acid changes Asp87Gly and Ser83Leu in GyrA. Ten isolates (43.5%) showed combinations of PMQR genes and QRDR mutations. Furthermore, the macrolide resistance genes *mph*(A) or *mph*(B) were found together in 13% of the sequenced genomes and, lastly, 8.7% carried the phenicol resistance gene *catA1*.

**Figure 3:**
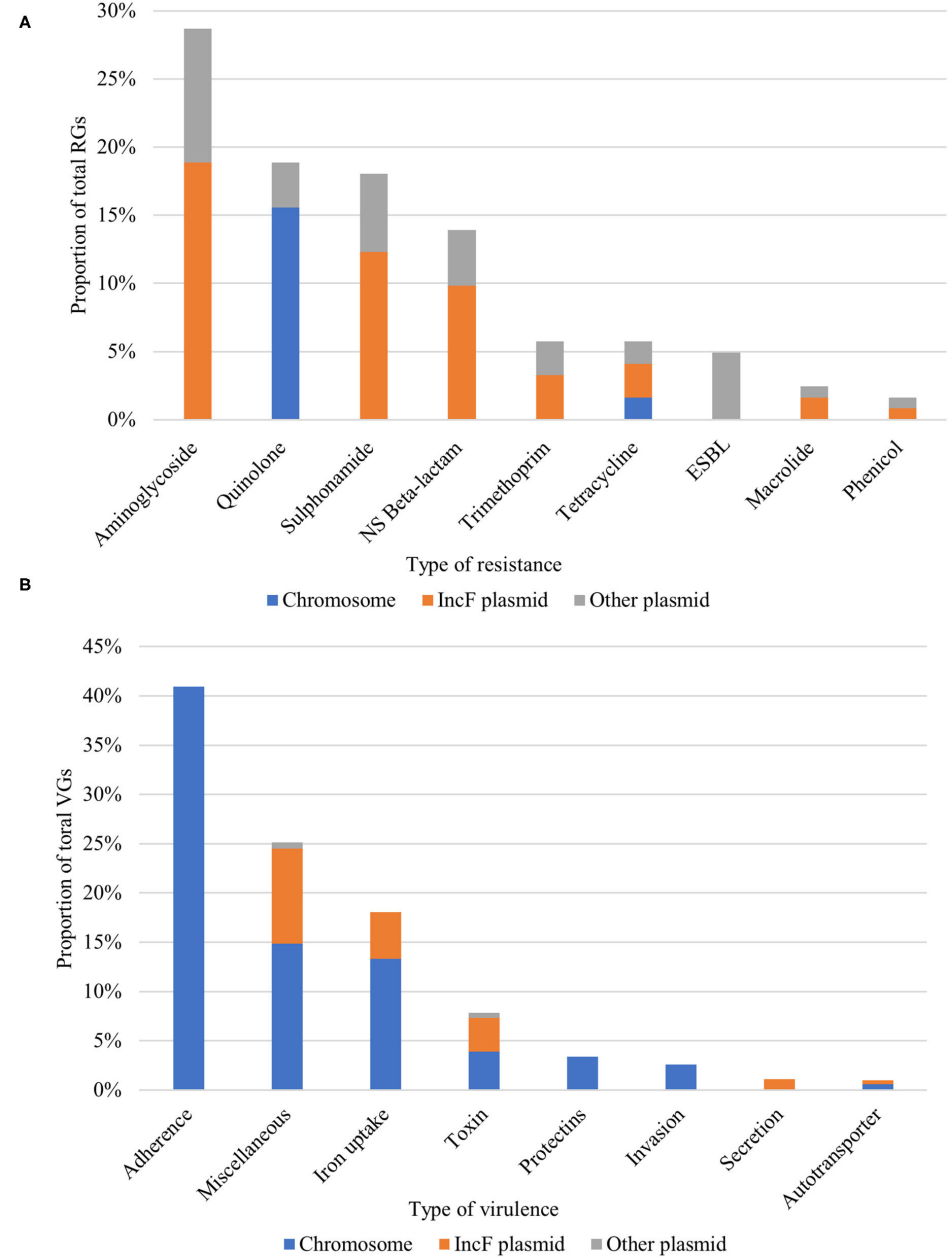
Proportion and genomic localization of **(A)** resistance genes (RGs) and **(B)** virulence genes (VGs). NS Beta-lactam, Narrow-spectrum beta-lactam; ESBL, Extended-spectrum beta-lactam.

We analyzed the APEC plasmids with ResFinder to elucidate the genomic localization of all RGs. Interestingly, almost half (49.18%) of RGs were located on IncF plasmids, and one-third of them (33.61%) was found on other non-IncF plasmids. The majority of RGs belonging to the most prevalent antimicrobial classes (aminoglycosides, sulphonamides, beta-lactams, and trimethoprim) were located on IncF plasmids. Conversely, the RGs conferring resistance to the critically important ESBLs and quinolones were located on non-IncF plasmids and the chromosome (Figure 3A).

### Virulence Genes

Overall, APEC subject to WGS carried virulence genes (VGs) belonging to 113 different VG-groups and individual isolates carried an average of 71 VGs (min = 57, max = 82). A total of 53 VGs were present in more than 90% of the isolates. These included the *fimD* fimbrial gene, which was also the most prevalent VG in our collection, flagellar proteins of the *fli* family, the *hlyE* and *hlyF* toxins and the metal ion binding gene *sitA*. Adherence factors were predominant (40.9% of total VGs) and an average of 30 adherence-related VGs (min = 22, max = 38) per isolate was detected (Figure 3B). The *eaeh* attaching and effacing gene was observed in the majority (95.6%) of sequenced APEC. About one-quarter (25.39%) of total VGs were of miscellaneous function, including hallmark APEC VGs such as the increased serum survival *iss* and the outer membrane protease *ompT* genes, both present in 95.6% of APEC. The third most abundant category consisted of genes responsible for iron uptake/transport (17.87% of VGs). Here, we found genes implicated in the virulence of ExPEC, APEC and UPEC, such as the aerobactin receptor *iutA* and siderophore receptor *iroN* (87% of APEC, each). Additionally, two-thirds of APEC carried the *fyuA* (yersiniabactin siderophore system). The rest of the virulence classes (toxins, protectins, autotransporters, invasion, and secretion genes) individually made up less 10% of the APEC VG content, they included however important VGs like the ExPEC marker *kpsMII* capsule (protectin) and the *hly*-like toxins. Moreover, sequenced isolates were assigned to pathotypes based on the previously defined criteria of VG content (29, 30). As expected, the majority of isolates (91.3%) not only fulfilled the minimum APEC predictors (positive for ≥2 of *iutA, hlyF, iss, iroN* and *ompT*) but possessed all five genes except for one isolate, which had four. For this latter subset of isolates (*n* = 22), 38% were additionally characterized as ExPEC (positive for ≥ 2 of *pap* (P fimbriae), *sfa*/*foc* (S/F1C fimbriae), *afa*/*dra* (Dr. binding adhesin), *iutA* and *kpsMTII*, and/or UPEC (positive for ≥2 of *chuA* (heme uptake), *fyuA*, vat (vacuolating toxin), and *yfcV* (adhesin). While the majority of VGs were located on the chromosome, IncF plasmids harbored all aforementioned pathotype-defining VGs and, conversely, only a small fraction of VGs were found on non-IncF plasmids (Figure 3B). APEC (*n* = 8) and AFEC-ESBL (*n* = 19) sharing the same STs and flocks of origin, showed a comparable number of VGs (mean = 88, min = 64, max = 102, and mean = 87, min = 65, max = 103, respectively). Interestingly, all but one AFEC-ESBL isolate possessed the minimum APEC predictors. Hierarchical clustering based on the presence/absence of VGs showed a strong correlation between VG profiles, STs, and phylogroups (Supplementary Figure 2).

### SNP-Based Phylogenetic Analysis

**Figure 4:**
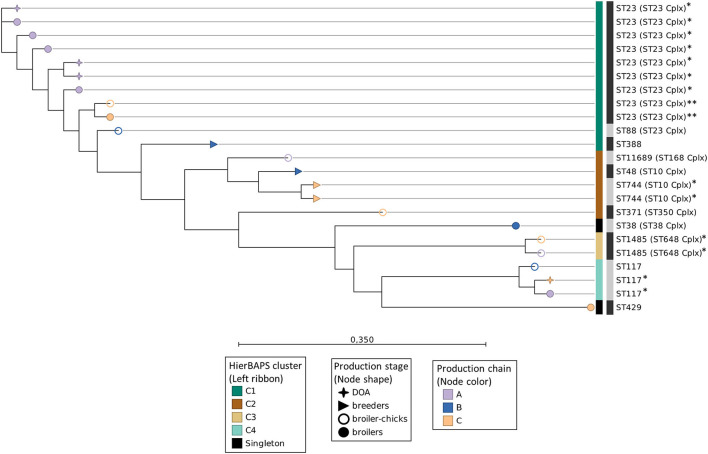
Maximum-likelihood SNP-based phylogeny of APEC whole-genome sequences. Nodes' shape and color indicate the broiler production stage and production chain, respectively. The left, colored ribbon specifies the hierBAPS clusters. The right ribbon with alternating dark and light gray segments, marks different STs. Isolates belonging to the same ST and indicated with the same number of asterisks (*) differed by <50 SNPs. Scale bar refers to the branch lengths, which are measured in the number of substitutions per site.

Genomes were mapped against the reference genome of *E. coli* APEC O1 (Accession no. NC_008563.1). All isolates covered at least 68.9% of the reference genome sequence, resulting in a core genome alignment of 3.5 Mbp and the identification of 112,928 informative SNPs. Cluster analysis with hierBAPS revealed the presence of four clusters (Figure 4). The examined isolates clustered primarily in accordance to their ST and subsequently to their ST complex. Additionally, each of the four most dominant STs (ST23, ST117, and ST1485) was assigned to an independent cluster. In the ST23 APEC of cluster C1, evidence of clonal transmission (0–50 SNPs) (9, 31) was found as seven isolates of chain A and two isolates of chain C had median pairwise differences (MPDs) of four and two SNPs, respectively, while a divergence of 1,073 SNPs between these two subsets was also observed (Figure 4). Additional evidence of clonal transmission events was identified in the isolates of ST744 (cluster C2), ST1485 (cluster C3), and ST117 (cluster C4) (Figure 4). We conducted an additional SNP-based phylogenetic analysis of APEC (*n* = 9) and AFEC-ESBL (*n* = 18) isolated from the same flocks and having the same STs. Interestingly, a close phylogenetic relationship among these isolates was revealed while five of the APEC were found to be clonally related (<50 SNPs) to AFEC of the same ST (Supplementary Figure 3). These five APEC were also 3GC-resistant (out of the 10/153 found in total) and shared their ESBL/pAmpC genes with the respective AFEC isolates. Conversely, the 3GC-susceptible APEC (*n* = 4) were more divergent (Supplementary Figure 3).

### IncF Plasmid Phylogenetic Analysis

APEC isolates analyzed by NGS carried on average two plasmids (min = 1, max = 5) belonging to eleven different plasmid replicons. All APEC were found to carry an IncF plasmid, accounting for 42% of identified replicons, while the other dominant replicons were Col-like (15%) and the IncI1-Iγ (9%). The most common IncF replicon sequence type (RST) was the F18:A-:B1 (52.2%) followed by the F18:A5:B1 (21.7%) in addition to three other RSTs. In all but three IncF plasmids, the ColV/BM colicins were identified. The APEC IncF plasmidome analyzed with PIRATE comprised 488 unique genes, with an average of 143 genes per plasmid (min = 89, max = 201). These included 15 genes present in at least 95% of the dataset, which formed the core IncF plasmidome, while the remaining and larger proportion of genes constituted the accessory IncF plasmidome. In the core region, we found the *repA* replication genes of the RepFIIA and RepFIB replicons, genes of the *tra* conjugative transfer locus (*traA, traE, traK* and *traX*), as well as nine additional genes of known (six) and unknown (three) function, leading to a core genome alignment of 5.5 Kbp. In the phylogenetic analysis, clustering of closely related IncF backbones corresponded to four main and one singleton RSTs (Supplementary Figure 4). Except for cluster C4, which included solely the F18:A-:B16 plasmids of ST744, the clusters comprised closely related plasmids of diverse STs and/or ST clonal complexes, suggesting horizontal transmission events via plasmid exchange in broiler production.

## Discussion

A high prevalence of colibacillosis across the broiler production pyramid was found in this study, with approximately one in three deaths being attributable to APEC-related infections. The highest APEC burden was observed at the top of the pyramid; however, the number of breeder samples was too small to allow for strong conclusions on the contribution of colibacillosis to the sampled breeders. Regardless of this limitation, previous studies indicated that the *E. coli* salpingo-peritonitis syndrome accounted for 34–52% of breeder losses (32, 33), a range that is within our confidence interval for this production stage. Considering the high value of breeders compared to the fattening broilers, the economic impact of this disease syndrome can be severe, costing about €1.87 per housed hen (33). In subsequent production stages, the occurrence of colibacillosis ranged from 34% in broiler chicks to 53.3% in DOA birds. Although these numbers are comparable with other investigations for end-cycle broilers (34), the occurrence of colibacillosis in broiler chicks might be considered low given that APEC infections resulting in severe omphalitis and septicemia can account for up to 70% of deaths in broiler chicks (7). An explanation for this finding may be related to the significantly increasing frequencies of phylogroups C and F along the production chain, which displaced the predominant one in broiler chicks, i.e., phylogroup A. More specifically, Logue et al. (35) found that the revised Clermont phylogenetic typing method provides a much more refined classification of commensal and pathogenic *E. coli* compared to older schemes that correlates closely with phylogenetic assignment and pathogenicity. In this context, the significant shifts in phylogroup composition can be interpreted as an increasing level of the APEC pathogenic potential along the production pyramid, moving from the commensal-like, opportunistic pathogens of phylogroup A in broiler chicks, to the highly pathogenic C and F groups in the end-cycle and DOA broilers (35), thus providing an explanation for the high mortality rates in end-cycle broilers. Our findings are further supported by the comparison of phylogroup distributions between APEC and AFEC, which showed a significant enrichment of phylogroup C in the APEC population (Supplementary Figure 1). A high mortality rate in the last stages of the production cycle is particularly problematic, as it can have a substantial negative impact on the feed conversion ratio and amount of meat available for sale (36).

We used the PSI with the phylogroup-serotype feature as input, to quantify the shifts of the APEC population across subsequent production stages. Notwithstanding the overall diversity in our collection, we found that at least part of the APEC genotypes among broiler chicks and end-cycle broilers overlaps, suggesting a persistence of certain APEC subpopulations in the production chain. These findings were reflected in our MLST analysis; first, we showed that the phylogroup-serotype feature can be used as an adequate indicator of APEC divergence in different sampling points, since different combinations were largely assigned to unique STs (Supplementary File 1). Second, in spite of the large phylogenetic diversity at the MLST level, APEC could be organized in four clusters of closely related STs that were dispersed over different production stages (Figure 2). Furthermore, at the highest resolution (WGS level), we obtained evidence of clonal transmission events within the APEC clusters. The identification of closely related APEC in different production chains and/or distantly located farms of the same chain, corroborates similar findings of previous studies (37, 38), indicating the potential to mitigate at least part of the APEC problem with interventions that target indirect transmission routes (e.g., farm-to-farm transmission), via stringent biosecurity controls in vehicles, equipment and personnel, which are usually shared in integrated production systems (39).

We screened all APEC isolates for resistance to 3GCs, which are critically important antimicrobials in human medicine (40). The isolate prevalence of 3GC resistance in APEC (6.5%) was comparable to the one found for commensal AFEC isolated from the same broiler flocks (7.8%) in a previous study (10), suggesting similar diffusion rates of 3GC determinants between the APEC and AFEC population in the studied broiler flocks. Moreover, the majority (86%) of APEC were MDR, while 65.2% had resistance to at least one critically important antimicrobial (3GCs, quinolones and macrolides) (Figure 3A). Furthermore, the SNP-based phylogenetic analysis of the APEC and AFEC-ESBL belonging to the same ST, showed that the majority (56%) of these APEC isolates were 3GC-resistant and clonally related to AFEC-ESBL, whereas 3GC-susceptible APEC were more divergent (Supplementary Figure 2). To our knowledge, this is the first description of a direct clonal relationship amongst 3GC-resistant *E. coli* isolated concurrently from the same flocks as commensals from the cloaca and as pathogens from tissue samples of dead broilers. Here, we showed that at least part of the APEC is directly linked to and therefore may have its origins in the commensal ESBL *E. coli* population.

In the virulence analysis, we observed a rich and diverse armament of VGs with various functions (Figure 3B). Of interest was the ubiquitous presence (95.6% of sequenced APEC) of the *eaeh* attaching and effacing gene, which has been demonstrated to play a central role in the pathogenesis of enterotoxigenic *E. coli* (41), whereas two-thirds of APEC carried the *fyuA* gene, which has been identified as an independent predictor of severe ExPEC infection (42). Moreover, we showed that apart from the basic APEC repertoire (30), isolates possessed combinations of VGs that assigned them to the non-avian pathotypes ExPEC and UPEC, which, according to a recent murine sepsis modeling study, have been validated as independent predictors of experimental virulence among fecal and clinical isolates (42). Considering the enriched resistance and virulence profile of the studied APEC isolates, as well as the links between resistant ExPEC infections in avian and human hosts that have been suggested previously (6, 43), the zoonotic potential of the APEC population in broilers should not be overlooked and be investigated further. Additionally, the predominant ST23 and ST117 of our APEC collection have avian species and humans as their major hosts according to the Enterobase database (data not shown) and have been previously implicated in human extraintestinal infections (6, 44, 45). The VGs comparison analysis displayed that the vast majority (94%) of the AFEC-ESBL isolates included in this study carried all five VGs used as APEC predictors, which is in agreement with previous studies suggesting that the broiler gut is enriched with “potential APEC,” i.e., commensal strains that carry numerous APEC related determinants (8, 34). Hierarchical clustering analyses based on 294 VGs showed that APEC and AFEC-ESBL belonging to the same ST and phylogroup clustered together, suggesting the presence of ST- or phylogroup-specific VGs, which might contribute to the predisposition of certain *E. coli* phylogroups to extra-intestinal pathogenicity.

In conclusion, it is important to stress the role of IncF plasmids. Our findings corroborate previous studies showing extensive IncF plasmid plasticity, specifically a small plasmid backbone consisting of basic functionality genes and a versatile accessory genome (46). Although the severity of infection is likely to be linked to a mixture of chromosomal and plasmidic genes with various functions (5) and no specific gene or set of genes have been proven to play an independent role in the development of colibacillosis in poultry, it is beyond doubt that IncF plasmid carriage is an important factor in the emergence of pathogenicity in *E. coli* (5, 8, 42). Here, using WGS data and plasmid reconstruction, we showed that while IncF plasmids carry a small fraction of the APEC virulome (Supplementary Figure 4), the VGs carried by IncF plasmids are fundamental for the manifestation of the APEC pathotype (42, 47) and thus HGT via plasmid exchange can promote pathogenicity in strains that are at the boundary between commensalism and the pathogenic state (47).

## Data Availability Statement

The datasets presented in this study can be found in online repositories. The names of the repository/repositories and accession number(s) can be found below: NCBI SRA; PRJNA745070.

## Ethics Statement

Ethical review and approval was not required for the animal study because the present study did not involve any invasive procedures. The animals subject to post-mortem examination died spontaneously and were collected on the first daily welfare walk conducted by farmers.

## Author Contributions

AP and IA designed the study. AP, IA, and AL acquired the data. IA, AL, and LM-G performed the data analysis and interpreted the data. IA prepared the first draft of the manuscript. AP, AL, LM-G, IA, and ÖY read, contributed to, and approved the final manuscript. All authors contributed to the article and approved the submitted version.

## Funding

This work was supported by the University of Padua [Fondi Investimento Strategico di Dipartimento (SID), Anno 2016-prot. BIRD167540].

## Conflict of Interest

The authors declare that the research was conducted in the absence of any commercial or financial relationships that could be construed as a potential conflict of interest.

## Publisher's Note

All claims expressed in this article are solely those of the authors and do not necessarily represent those of their affiliated organizations, or those of the publisher, the editors and the reviewers. Any product that may be evaluated in this article, or claim that may be made by its manufacturer, is not guaranteed or endorsed by the publisher.

## Supplementary Material

Supplementary Figure 1 Comparison of phylogroups' distribution between avian fecal *E. coli* (AFEC) and avian pathogenic *E. coli* (APEC) in the broiler production pyramid. *P* ≤ 0.05 between compared groups (indicated with brackets) denote significant differences in the enrichment of phylogroups between the two *E. coli* populations.

Supplementary Figure 2 Dendrogram depicting the hierarchical relationship between AFEC-ESBL and APEC inferred by a presence/absence distance matrix of virulence genes (VGs). Nodes' shape and color indicate the broiler production stage and production chain, respectively. The left, colored ribbon specifies the presence of ESBL/pAmpC genes. The right ribbon marks the different *E. coli* phylogroups. Gray and black text labels indicate AFEC and APEC isolates, respectively.

Supplementary Figure 3 Maximum-likelihood SNP-based phylogeny of APEC and AFEC-ESBL whole-genome sequences. Nodes' shape and color indicate the broiler production stage and production chain, respectively. The left, colored ribbon specifies the presence of ESBL/pAmpC genes. The right ribbon with alternating dark and light gray segments, marks different STs. Isolates belonging to the same ST and indicated with an asterisk (^*^) differed by <50 SNPs. Gray and black text labels indicate AFEC and APEC isolates, respectively. Scale bar refers to the branch lengths, which are measured in the number of substitutions per site.

Supplementary Figure 4 Maximum-likelihood core-genome-based phylogeny of IncF plasmid sequences predicted with MOBsuite. Nodes' shape and color indicate the broiler production stage and production chain, respectively. The left, colored ribbon specifies the hierBAPS clusters. Labels on the right denote the different STs. Scale bar refers to the branch lengths, which are measured in the number of substitutions per site.

Supplementary File 1 Metadata of APEC isolates included in this study.
