# Supplementary figures and images for: Occurrence of Colibacillosis in Broilers and Its Relationship With Avian Pathogenic Escherichia coli (APEC) Population Structure and Molecular Characteristics
Source: Front Vet Sci. 2021 Sep 8;8:737720. doi: 10.3389/fvets.2021.737720 (PMC8456121; doi:10.3389/fvets.2021.737720)

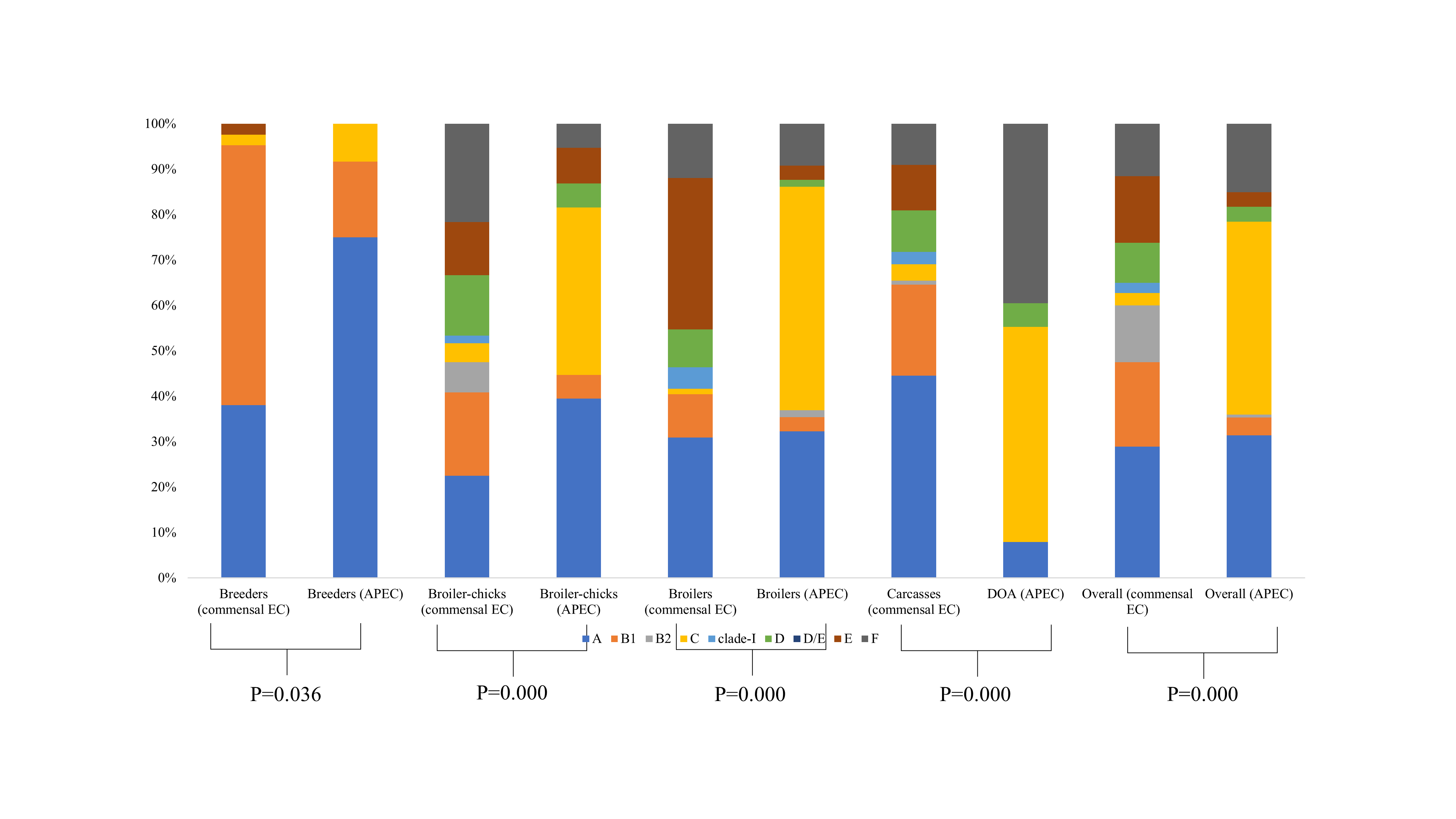

Supplement: Supplementary Figure 1 — Comparison of phylogroups' distribution between avian fecal E. coli (AFEC) and avian pathogenic E. coli (APEC) in the broiler production pyramid. P ≤ 0.05 between compared groups (indicated with brackets) denote significant differences in the enrichment of phylogroups between the two E. coli populations. [file Image_1.TIF]

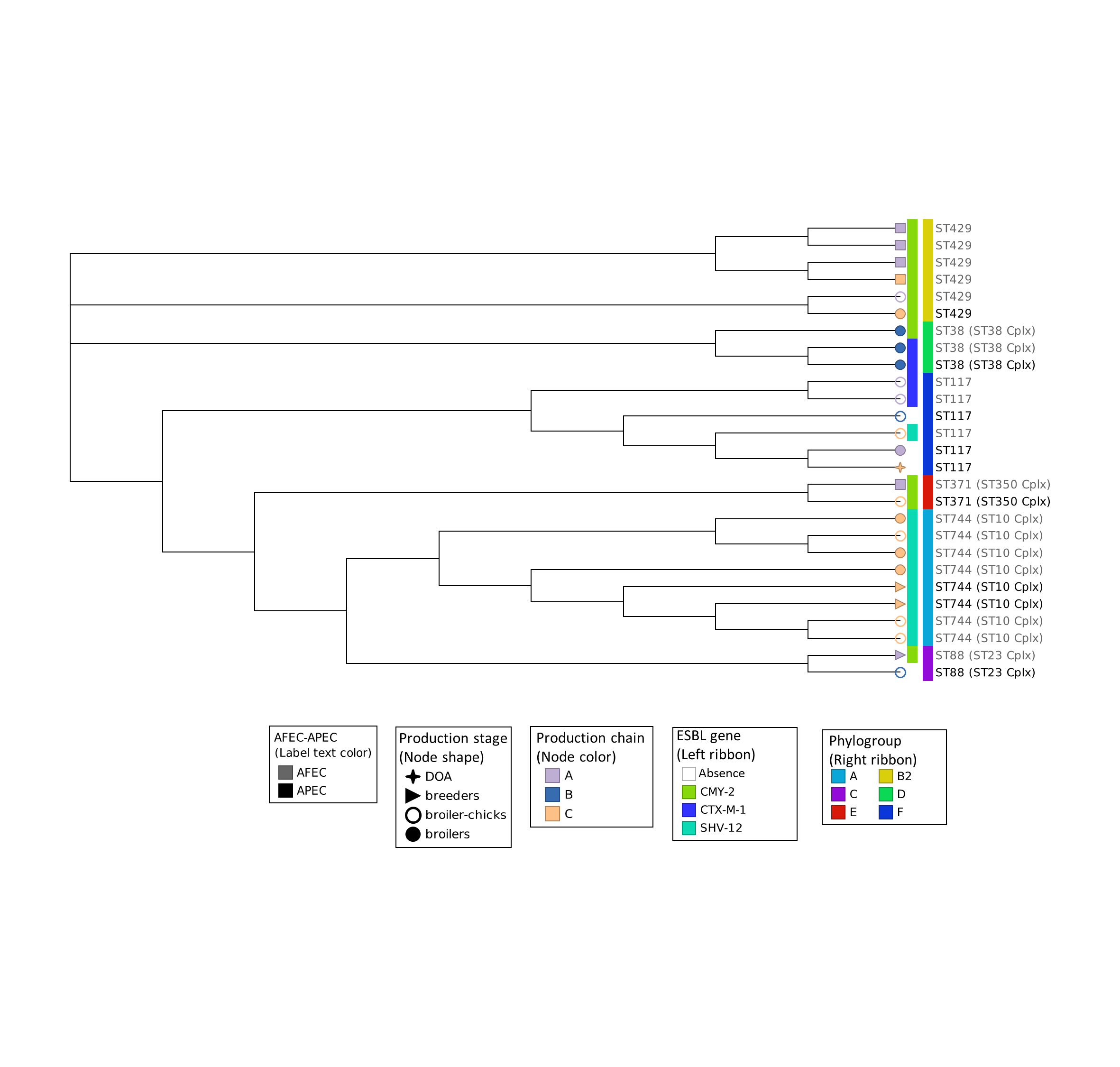

Supplement: Supplementary Figure 2 — Dendrogram depicting the hierarchical relationship between AFEC-ESBL and APEC inferred by a presence/absence distance matrix of virulence genes (VGs). Nodes' shape and color indicate the broiler production stage and production chain, respectively. The left, colored ribbon specifies the presence of ESBL/pAmpC genes. The right ribbon marks the different E. coli phylogroups. Gray and black text labels indicate AFEC and APEC isolates, respectively. [file Image_2.TIF]

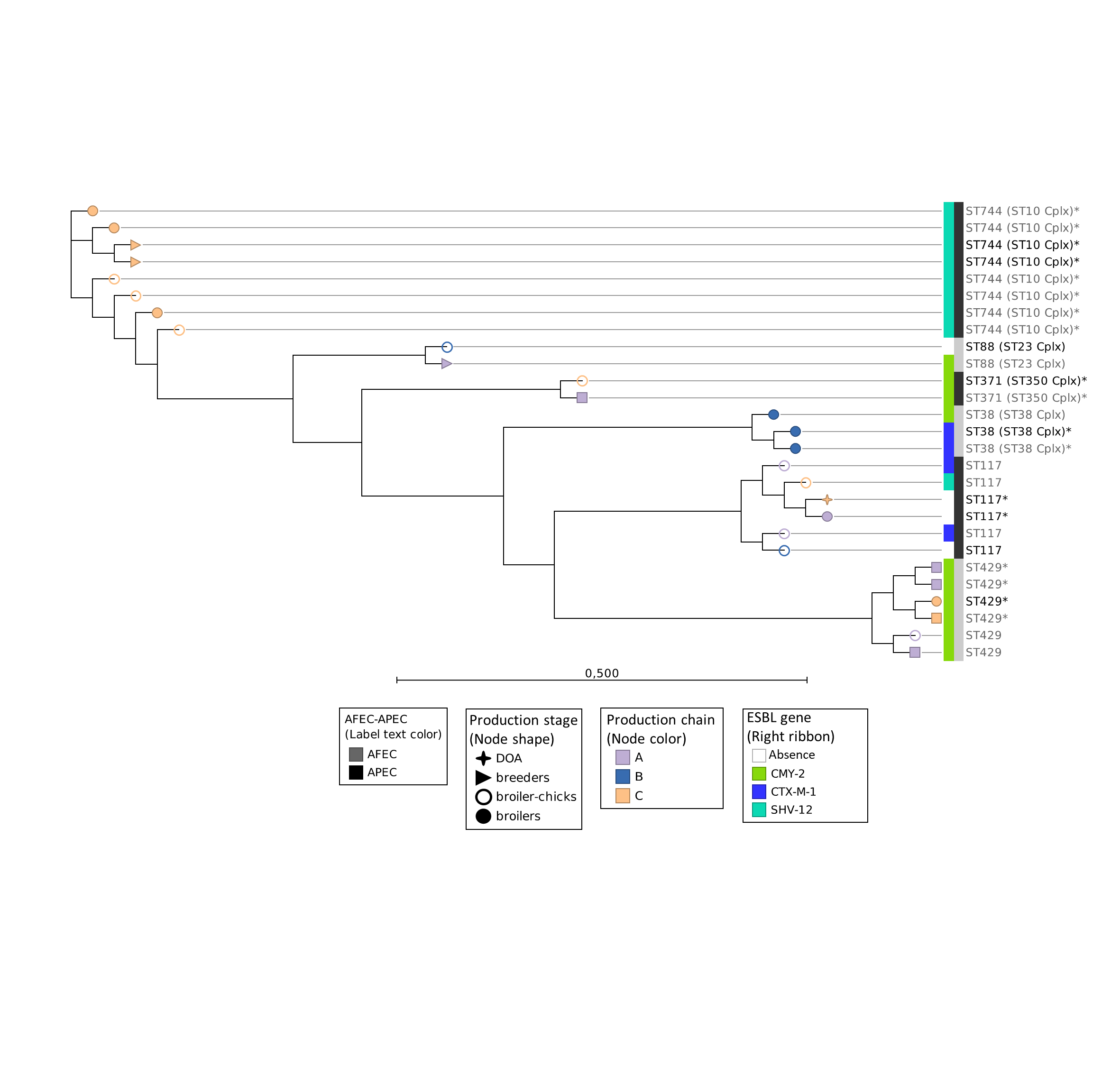

Supplement: Supplementary Figure 3 — Maximum-likelihood SNP-based phylogeny of APEC and AFEC-ESBL whole-genome sequences. Nodes' shape and color indicate the broiler production stage and production chain, respectively. The left, colored ribbon specifies the presence of ESBL/pAmpC genes. The right ribbon with alternating dark and light gray segments, marks different STs. Isolates belonging to the same ST and indicated with an asterisk (*) differed by <50 SNPs. Gray and black text labels indicate AFEC and APEC isolates, respectively. Scale bar refers to the branch lengths, which are measured in the number of substitutions per site. [file Image_3.TIF]

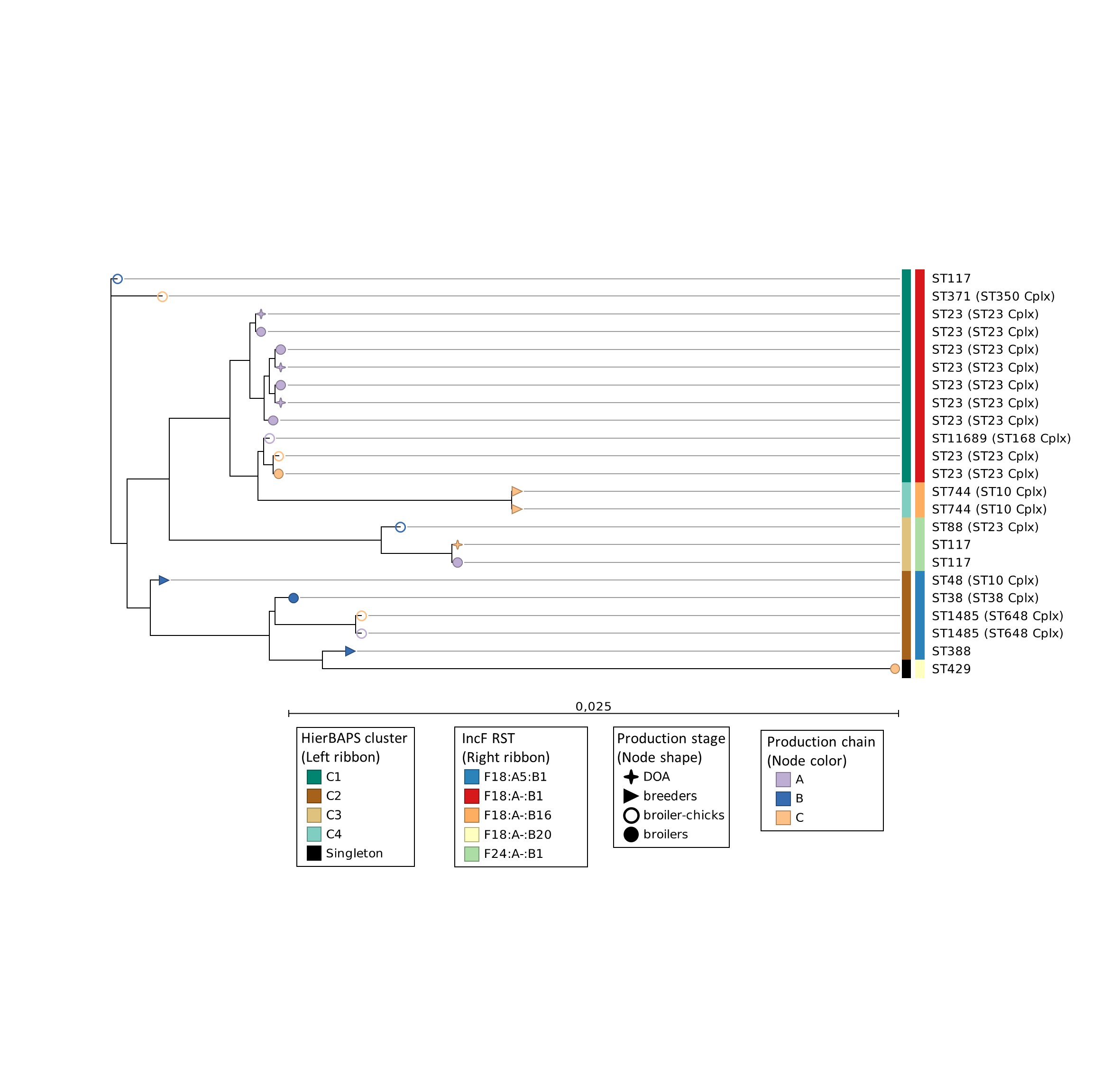

Supplement: Supplementary Figure 4 — Maximum-likelihood core-genome-based phylogeny of IncF plasmid sequences predicted with MOBsuite. Nodes' shape and color indicate the broiler production stage and production chain, respectively. The left, colored ribbon specifies the hierBAPS clusters. Labels on the right denote the different STs. Scale bar refers to the branch lengths, which are measured in the number of substitutions per site. [file Image_4.TIF]
